# Selections and Abstracts

**Published:** 1916-08

**Authors:** 


### SELECTIONS AND ABSTRACTS

### UNITED STATES PUBLIC HEALTH SERVICE.

Washington, August 14, 19116.

Congress has recently made an appropriation for 33 additional Assistant Surgeons in the United States Public Health Service. These officers are commissioned by the President, and confirmed by the Senate. The tenure of office is permanent, and successful candidates will immediately receive their commissions.

After four years' service, assistant surgeons are entitled to examination for promotion to the grade of passed assistant surgeon. Passed assistant surgeons after twelve years' service are entitled to examination for promotion to the grade of Surgeon.

Assistant Surgeons receive $2,000, passed assistant surgeons $2,400, surgeons $3,000, senior surgeons $3,500, and assistant surgeon-generals $4,000 a year. When quarters are not provided, commutation at the rate of $30, $40, and $50 a month, according to the grade, is allowed.

All grades receive longevity pay, 10 per cent. in addition to the regular salary for every five years up to 40 per cent. after twenty years' service.

Examinations will be held every month or so in various cities, for the convenience of candidates taking the examination. Further information will be furnished by addressing the Surgeon-General, United States Public Health Service, Washington, D. C.

---

### UNITED STATES PUBLIC HEALTH SERVICE.

It is a remarkable fact, confirmed by many observations, that many physicians who have devoted considerable labor to the study of a particular disease have themselves died of that disease. One of the most interesting examples is that of John Daniel Major, born August 16, 1634, in Breslau, a physician and naturalist of no mean ability. Bitten early by the wanderlust, he studied at Wittenburg, took courses at many of the schools in Germany, and finally went to Italy where he received the degree of doctor of medicine at Padua in 1660. Returning to his own country, he resided for a short time in Silesia, and in 1661 married at Wittenburg, Margaret Dorothy, a daughter of the celebrated Sennert. The following year, his young wife was stricken with plague and died after an illness of eight days. Distracted by his loss, Major wandered up and down Europe studying plague wherever he found it in the hope that he might discover a cure for the disease which had bereaved him. Spain, Germany, France and Russia were visited by him. He settled in 1665 in Kiel, where he was made professor of botany and the director of the botanical gardens. He made frequent voyages, however, always in quest of the remedy for plague. Finally in 1693, he was called to Stockholm to treat the queen of Charles the Eleventh, then ill with plague.

But before he could render her any service, he contracted the disease and died on the third of August.

The bubonic plague of today is identical with the black death of the Middle Ages. Primarily a disease of rodents caused by a short dumb-bell shaped microscopic vegetable, the pest bacillus, it occurs in man in three forms; the pneumonic, which has a death rate of almost 100 per cent.; the septicaemic, which is nearly as fatal, and the bubonic in which even with the most modern methods of treatment the mortality is about 50 per cent. It is a disease of commerce, spreading around the globe in the body of the ship-born rat. It is estimated that every case of human plague costs the municipality in which it occurs at least $7,500. This does not take into account the enormous loss due to disastrous quarantines and the commercial paralysis which the fear of the disease so frequently produces.

The disease is now treated by a serum discovered through the genius of Yersin. This is used in much the same way as is diphtheria antitoxin.

Plague is transferred from the sick rodent to the well man by fleas. The sick rat has enormous numbers of plague bacilli in its blood. The blood is taken by the flea, which, leaving the sick rat, seeks refuge and sustenance on the body of a human being to whom it transfers the infection.

Since plague is a disease of rodents and since it is carried from sick rodents to well men by rodent fleas, safety from the disease lies in the exclusion of rodents, not only exclusion from the habitation of man but also from the ports and cities of the world. Those who dwell in rat-proof surroundings take no plague. Not only should man dwell in rat-proof surroundings, but he should also live in rat-free surroundings. The day is past when the rodent served a useful purpose as the unpaid city scavenger. Rats will not come where there is no food for them. Municipal cleanliness may be regarded as a partial insurance against plague. The prayer that no plague come nigh our dwelling is best answered, however, by rat-proofing the habitations of man. Modern sanitary science has evolved a simple and efficient weapon against the pestilence which walketh in darkness and striketh at noonday, and the U. S. Public Health Service has put this knowledge into practical operation and thus speedily eradicated plague wherever it has appeared in the United States.

---

### AMERICAN PROCTOLOGY SOCIETY.

EIGHTEENTH ANNUAL MEETING, HELD AT DETROIT, MICH., JUNE 11 AND 12, 1916.

The President, T. Chittenden Hill, M. D., Boston, Mass., in the chair.

Officers elected for the ensuing year:

President: Alfred J. Zobel, M. D., San Francisco, Cal.

Vice-President: Granville S. Hanes, M. D., Louisville, Ky

Secretary-Treasurer: Collier F. Martin, M. D., Philadelphia, Pa.

Executive Council:

1. T. Chittenden Hill, M. D., Boston, Mass.
2. Alfred J. Zobel, M. D., San Francisco, Cal.
3. Wm. M. Beach, M. D., Pittsburg, Pa.
4. Collier F. Martin, M. D., Philadelphia, Pa.

The place of meeting for 1917 will be New York, N. Y. Exact date and headquarters will be announced later.

The following were elected Associate Fellows of the Society: W. Oakley Hermance, M. D., 2040 Pine St., Philadelphia, Pa.; Geo. B. Moreland, M. D., F.A.C.S., 303 Second National Bank Building Pittsburg, Pa.

### WHY PROCTOLOGY HAS BEEN MADE A SPECIALTY.

By T. Chittenden Hill, M. D., of Boston, Mass.

In this address Dr. Hill calls particular attention to the inadequate treatment that rectal fistula receives at the hands of the general surgeon. He claims that the general surgeon "has never taken the pains to learn the underlying principles of a fistula operation, nor has he the requisite skill, experience or inclination to carry out the necessary steps in the post-operative treatment of these cases, to bring them to a successful conclusion."

While in London there are two hospitals devoted to the ex clusive treatment of disease of the rectum, Hill feels that better results can be obtained by establishing special departments in our large general hospitals. He urges that Proctologists be appointed to all general hospitals. The many advantages of staff association, consultations, etc., in which proctology touches on the work of men in other fields, would prove of mutual benefit.

He believes that in the near future a fifth year will be added to the present four-year medical course. This fifth year will probably be devoted to the medical specialties and proctology should be included among them. The undergraduate certainly should have the chance to acquire reasonable proficiency in the newer methods of examination and treatment of rectal disease.

Dr. Hill also presents a formal paper for the consideration of the members of the Society under the title of

### PROLAPSUS ANI IN ADULTS.

By T. Chittenden Hill, M. D., of Boston, Mass.

The theory is advanced that all cases of procidentia recti are the result of neglect or improper treatment of what was in the beginning a simple form of mucous membrane prolapse. Correction of the condition early may prevent serious infirmity later in life.

He describes at length an operation modified after that of the late Mr. Goodsall, of London, England.

In this operation he employs a multiple suture. He advises removing the excess of tissue distal to the ligature.

The operation is performed under local anesthesia and is advised for patients of all ages. It is particularly suitable for use in prolapse of the age.

The author claims that the operation is painless, short and easily performed. There is absence of hemorrhage and the end results are satisfactory.

### THE POST OPERATIVE TREATMENT IN RECTAL SURGERY.

By W. H. Stauffer, M. D., of St. Louis, Mo.

This paper is based upon a review of over 25,000 rectal cases treated, of which 1,500 were operative. Four hundred of these cases had been operated upon previously by approved method by other surgeons.

There are two reasons for these 400 secondard operations. First: Not selecting the operation indicated by the pathology. Second: Improper post-operative attention.

In selecting an operation or treatment the following requirements must be met. First: Complete restoration of functions. Second: Time required for cure. Third: Pain produced.

Unsatisfactory results—complete or partial incontinence often are caused by needless traumatism. He does not believe in divulsion. Division of nerves causes sensory disturbances.

Incontinence may be due to fistula operation. Believes that where the fistula opens more than two inches above the sphincter the two-step operation is indicated.

In dealing with malignancy he mentions the operation of Evans as producing the least mutilation and disturbance of function in selected cases.

Operations should only be performed after a definite diagnosis has been made.

It is insisted that the best results are obtained by proper diagnosis, careful preparation, appropriate operation, and careful after-treatment. The surgeon should always make the first dressing and should always inspect the operative field daily. The patient should be kept under observation until recovery is assured.

### PHOTOGRAPHY FOR RECORD AND TEACHING.

By Collier F. Martin, M. D., of Philadelphia, Pa.

Martin draws attention to the fact that students may be better interested in a lecture if their attention be fastened by an appropriate picture or illustration. After experimenting with photographs or drawings, passed among his class, and also with charts hung on the walls he found that he could better interest the students with lantern slides thrown upon a screen. The darkness of the room tends to lessen the distraction and to encourage concentration. By having photographs of actual cases, as well as of the different steps in an operation, it was easy to interest the class and to explain far better than could be done even in a clinical lecture.

The equipment is briefly described and suggestions are given as to proper rendering of color values by the use of light-filters.

Attention is called to the necessity of proper exposure and lighting to give negatives with sufficient detail to properly show pathologic conditions. Such negatives only are useful for illustrations, record or lantern slides.

Many case histories are incomplete without a photograph to clarify the description.

Hints are given for copying, making line drawings, diagrams and classifications to produce lantern slides suitable for teaching.

It is suggested that every hospital have a department devoted to photography. This could easily be operated in conjunction with the x-ray department.

### ABSTRACT — SOME IMPORTANT PATHOLOGICAL CONDITIONS ABOUT THE RECTAL OUTLET: LANTERN SLIDE DEMONSTRATIONS.

By Granville S. Hanes, M. D., F.A.C.S., Louisville, Ky.

Tubercular ulcerations do not occur as frequently in the mucosa of the rectum and sigmoid as is generally believed. Amebic and various types of bacterial ulceration produce dysenteric symptoms that often lead to emaciation and exhaustion. Active tubercular ulceration is always accompanied by a decided increase in the temperature and pulse rate. These are not characteristics in other types of ulceration. In tubercular ulceration there is a history of constant and progressive symptoms while in amebic there is usually a history of improvement and relapses. Tubercular ulceration involving the rectum and sigmoid seldom yields to treatment. Amebic ulceration in this climate can be cured by one method or another.

Bacterial types of ulceration are usually very difficult to treat. Within the last two years I have found cauterization with the high tention electric spark to be a most valuable means of treatment.

Tubercular abscesses often occur about the rectum when patients otherwise show no evidence of tuberculosis. The abscesses and subsequent fistulae are characteristic in that there is a great tendency to undermining of the skin. The external openings are, therefore, large with a livid appearance of the surrounding cutaneous structures. They point to impending trouble which may be precipitated months or years hence. This being true it is of great importance that we direct the habits, hygiene, etc., of individuals thus afflicted.

Fistulae of long standing with one or more very small external openings with a history of an extensive abscess are very difficult to cure. From external evidences they appear to be very simple. Unusually the finger when introduced well into the rectum will be able to detect by careful palpation the hard indurated sinuses which often extend surprisingly high up by the rectum.

Internal fistulous openings rarely, if ever, perforate the rectal wall unless there is some pathology primarily in the rectal mucosa whereby its resistence is impaired. The internal openings of the fistulae are usually in the anal canal. The anal tissues are most always diseased before the abscess is formed, therefore, it is reasonable to suppose that the infection passes out through the diseased anal structures and is responsible for the abscess.

There are occasional fistulous tracts that extend up by the rectum to considerable heights and are very tortuous. It is difficult to follow these sinuses to their terminations when operating. When the wound heals and a small opening remains we may feel fairly certain that some part of the original fistula was not reached. It is then advisable to inject bismuth paste which will often effect a cure.

Pruritus ani is undoubtedly a local infection. The focus of the disease is below the pectinate line and at the anal margin. It has been my practice to remove the diseased tissues at the margin of the anis and from the emulsion of these diseased structures bacteria are cultivated and an autogenous vaccine administered to the patient. The operation with autogenous vaccine obtained in this manner give decidedly the best results.

### ABSTRACT OF PAPER—PRELIMINARY REPORT: ANATOMICAL AND BACTERIOLOGICAL FINDINGS OF THE ANORECTAL REGION.

Dr. J. Rawson Pennington, Chicago: This preliminary report is submitted in lieu of my paper on "Indications for Making a Rectal Examination."

Today the question of "focal infection" is uppermost in the minds of the medical profession. Much consideration has been given to practically every point in the body from which focal infections may emanate except that of the anorectal region.

Experimental investigations show that not only Crypts of Morgagni, but what appears to be diverticuli are found also in this region. The Medical Research Laboratory of Chicago, to whom specimens were submitted for examination, reports that these diverticuli are lined with stratified squamous epithelium. Also that streptococci, staphylococci, colon bacilli, and other bacteria were found in their tunics and sacs.

We have observed that local and constitutional diseases may be produced by injecting the various bacteria obtained from these diverticuli into animals.

I am investigating the value of these diverticula as points of focal infection and their role as causative factors in hemorrhoids, fistula, constipation, arthritis, endocarditis and other acute, and chronic, and local and constitutional infections.

### SOME OBSERVATIONS ON HERNIA IN RELATION TO INTESTINAL STASIS.

By William M. Beach, M. D., of Pittsburgh, Pa.

After reviewing the theories of Keith relative to nodal zones situated at different levels in the intestinal musculature the author says that:

1. We have tried to define intestinal stasis to be a physiologico-anatomic disturbance of peristalsis by an inhibiting influence through nodal zones of the myenterum, located in the aesophago-gastric junction, the duodeno-jejunal area, ileocaecal region and in the rectum. This demonstrated in the laboratory must be verified clinically.

2. Anatomic distortions, as kinks, adhesions, ptoses, etc., lead to stasis by disturbing the ganglia controlling peristalsis.

3. Hernia is a frequent manifestation of visceral displacement concomitant with stasis.

4. Long truss wearing with great pressure tends to rectal disease.

### ABSTRACT—INTESTINAL SYMPTOMS DUE TO ACHYLIA GASTRICA.

By Alois B. Graham, A. M., M. D., F.A.C.S.,
Clinical Professor of Proctology, Indiana University School of Medicine, Indianapolis, Indiana.

In 5758 patients presenting gastro-intestinal symptoms, and in every one of whom repeated gastric analysis were made, a diagnosis of Achylia Gastrica was made in 378. This is about 6.5 per cent., or a ratio of 1 to 5. 100 were males and 278 females. The youngest was 17 years, the oldest 73 years. 60 per cent. were between the ages of 40 and 60 years. In 90 per cent. the subjective symptoms were chiefly intestinal in character. The bowels were reported regular in 38; constipated in

112; loose (diarrhoea) in 142; irregular in 86. Diarrhoea was the most frequent symptom and was present in 37.5 per cent. of the cases. Description of three groups of cases. Description of the stools which were at times quite characteristic. Rectal symptoms rarely reported. Internal hemorrhoids found in every case. Rectal examination of no value, except that of exclusion, in determining the cause of the intestinal symptoms. In cases where constipation was chief symptom, there was not anything of special interest.

There was no return of the gastric secretion in any of the cases. The course of Achylia Gastrica is a protracted one. Under proper therapy the prognosis, as to fairly good health, is excellent.

Diet alone in the severe cases of diarrhoea was not successful. Astringents and intestinal irrigations were unsuccessful. Hydrochloric acid and pepsin in sufficient dosage is rational therapy and the only one which gave anything like satisfactory results. In some cases diet and hydrochloric acid failed. In these cases a nervous element was present as the administration of bromides in suitable dosage produced most excellent results.

Patients are comfortable as long as they continue treatment. If discontinued even for a brief period, there is a recurrence of the diarrhoea. These patients should be correctly informed as to the prognosis; namely, that as long as there is evidence of an absence of the gastric secretion, just so long must they adhere to a rigid diet and take hydrochloric acid and pepsin.

### OBSERVATIONS ON FISSURE IN ANO.

Rollin H. Barnes, M. D.,
Editor of The Proctologist and Gastroenterologist,
St. Louis, Missouri.

The author considers fissure as an ulcer and believes that traumatic cases are not true etiological factors in the production of this trouble, but that it is necessary that the tissues becomes inflamed and hence friale and easily torn in order that fissure be formed. He believes that catarrhal inflammatory conditions are frequently the result of an excessive carbohydrate diet and sometimes an excessive fat diet.

In the treatment of fissure he recommends palative treatment by correcting the diet with reference to the excesses of carbohydrates and fats and placing the patient on a proteid diet for a time. When operation is necessary he believes that the object should be drainage rather than paralyzing the muscular fibers. He also advocates the use of a small enema before defecation in order to avoid irritation from the stool. It is very important to keep the wound clean by hot sitz baths and the hot enema, in order that any foreign substance may not remain in the wound.

### ABSTRACT—MALIGNANT TRANSFORMATION OF BENIGH GROWTHS.

By Frank C. Yeomans, A. B., M. D., F.A.C.S.,

Adjunct Professor of Proctology, N. Y. Polyclinic Medical School and Hospital, New York City.

The benign tumors of the colon and rectum considered were of the polypoid type,—solitary polyp, multiple polyposis, multiple adenomata and villous tumor. All originate from the intestinal mucosa, are of the same histologic structure but differ in number, size, form and the relative amounts of glandular and fibrous tissue present.

The writer cites the theories of origin of multiple adenomata as advanced by Mayer, Liebert and Schwab and G. Houser and H. C. Ross's views on the formation of benign growths. Yeomans things these tumors inflammatory in character and notes the frequent history of colitis or dysentery in these cases, intestinal parasites as causal in others and the positive evidence of the role of irritation as furnished by therapy,—colonic lavage, or colostomy and irrigation benefitting some patients and curing others. He reports a case of multiple adenomata in a man, aged 30, colostomized in 1913, with marked benefit. Many tumors have disappeared, the remainder have retrogressed and the patient is working regularly. There is no evidence of malignant change.

That benign growth become malignant is beyond cavil but its cause involves the same enigma as the cause of cancer itself. The writer cites the work of neoplasms of Waldeyer, Adami, Cathcart and others, as well modern research on the transplantation of tumors and the parasitis theory of their origin. He concludes: "All that can be stated positively is that cancer begins as a small local process; that it excites no reaction in the blood whereby a diagnosis can be made; that the individual cancer cell is the parasite of cancer, and whatever eventually explains the origin of cancer will also explain the transformation of a benign into a malignant growth."

Yeomans reports the transformation of a simple adenoma into an adenocarcinoma in a man, aged 76, who had rectal bleeding of 8 years' duration, progressive contsipation and a tumor that in recent years could not be reduced within the rectum. The tumor, three and one-half by two inches, was attached just within the anal verge. It was removed under local anesthasia and both clinically and histologically was adenocarcinoma.

Villous tumor or adenoma tends to recur in malignant form so should be extirpated early, thoroughly and radically.

Multiple adenomata are the most important and serious type of benign growth of the intestine. Their usual site is the lower colon and rectum. Clinically they are malignant from diarrhoea, haemorrhage, etc., and if neglected over 40 per cent. become actually malignant. Improper local treatment, as snaring, curettage and cauterization is followed by malignant recurrence in a large proportion of cases.

The curative, operative procedure indicated is enterotomy, either in the colon above the growths, or in the terminal ileum when the entire colon is affected. If the tumors disappear, the enterotomy may be closed. If they persist, after prolonged irrigation and the patient's general condition warrants it, partial or total colectomy is indicated with implantation of the ileum low down into the sigmoid, the operation being performed either in one or preferably in two stages.

### THE TREATMENT OF HEMMORRHOIDS BY A NEW METHOD.

E. H. Terrell, M. D., Richmond, Va.

The author presents a simple, safe and efficient method o1 curing selected cases of Hemmorrhoids by the injection of quinine and urea solution. During the past two years 127 patients have been treated by this method with only one recognized failure. Injection of quinine and urea in solutions of from 5 per cent. to 20 per cent. strength produces starvation and atrophy of the hemorrhoids. The series reported includes only uncomplicated internal hemorrhoids. The results of the treatment of 127 patients justify conclusion that the method is simple, safe and effective in properly selected cases.

### ABSTRACT ON THE ETIOLOGY OF VACCINE TREATMENT OF PRURITUS ANI.

Louis J. Hirschman, Detroit, Mich.

Hirschman presented a preliminary report of his work on the bacteriology of pruritus ani as based on the original work of Murray at Syracuse. The work of H. C. Ward, bacteriologist, in conjunction with Hirschman's work shows that the streptococcus faecalis was present in the twenty-five cases, but the vaccine treatment in these cases, especially that of the autogenous vaccines, has resulted in important or systemic cure in but four cases, while the treatment of the surgical lesions present, or by dietary, or hygienic measures, has resulted in relief or cure of all the remaining cases.

### ABSTRACT OF PAPER ENTITLED FURTHER OBSERVATION ON PRURITUS ANI, ITS ETIOLOGY AND TREATMENT.

(A sixth report based on results of original research).

Dr. Dwight H. Murray, of Syracuse, N. Y., read the sixth annual report of his original research work on Pruritus Ani and Vulvae adding reports of 25 cases to the former series of cases, making 123, the bacteriology of which shows 95 per cent. of the cases a streptococcic infection as the etiology for these troublesome conditions. He stated that his claim, that the streptococcus fecalis is the etiology of Pruritus Ani, is now confirmed by leading physicians throughout the United States, who have been investigating the subject.

He finds from experience of this past year that far better results are obtained by the use of autogenous vaccines with more than 1000 million dead germs to 1 C. C.

He states that not one of the cases of Pruritus Ani and Valvae Pruritus Scroti in the 123 cases have had diabetes and, as a result of this, he questions very strongly whether diabetes is ever the cause of these conditions, unless as a complication, and under such condition there would be a general pruritic condition of the skin.

Last year, in his fifth report, he described cases of Pruritus Ani that did not show improvement under the administration of the autogenous, streptococcic vaccine. These cases were later found to have a staphyhococcic infection as a complication and when an autogenous staphylococcus vaccine was administered with the autogenous, streptococcic vaccine improvement resulted. He has found proof of this same condition during the past year and believes that these cases show a characteristic whitish appearance of the skin in spots, particularly around deep skin fissures.

He also found further proof of one of the conclusions, in a former paper, i. e., where there is a rectal pathology with Pruritus Ani, plus a skin infection, that an operation for relief of these conditions will cure the rectal pathology, but will not cure the Pruritus Ani. If the strept ococcic skin infection does not exist the operation will be very sure to cure Pruritus Ani.

During the six years that Dr. Murray has been doing this work he has never had as prompt and satisfactory results from treatment as during the past year. In his report of the present condition of patients treated during the past five years, he shows that practically all of the patients have retained a part of the benefit originally received and a large majority of them consider themselves cured. Time will give the proof of this.

While some of the cases still have a little itching from time to time, they state that it is very easily controlled, by simple methods.

Dr. Murray is more firmly convinced than ever that operations for the cure of Pruritus Ani, such as Balls' operation and modifications of it, are absolutely contradicted and should never be performed.

### "ANO—RECTAL INJURIES."

Samuel Goodwin Gaut, M. D., L. L. D.

The author stated that while the rectum is protected by the buttocks, and bony structures, it is frequently injured by external trauma, expulsion of hardened feces, and foreign bodies, swallowed or introduced through the anus, such wounds being contused, lacerated, incised or perforated.

Laceration of one or all of the rectal coats, results from careless examinations, introduction of imperfect syringe nozzles, bougies, proctoscopes, or other instruments.

Perforating wounds are caused by bullets, knife thrusts, and pointed objects that have been swallowed, or introduced into the rectum, except when due to specific ulcers or cancer.

Recently many pneumatic rectal ruptures, the result of compressed air introduced through the anus, in a spirit of fun, have been reported.

The injection of carbolic acid, into hemorrhoids is responsible for extensive ano-rectal injuries.

Symptoms: The chief manifestations of superficial ano-rectal injuries, are bleeding, sphincteralgia, frequent micturition, and painful defecation; symptoms that are exaggerated, when wounds are extensive.

Infected wounds are characterized by a chill, temperature, throbbing pain, swelling, and a thick yellow discharge.

In extensive injuries of the upper rectum, hemorrhage is profuse. There is shock, the patient collapses, and soon exhibits symptoms of peritonitis, when the peritomeum is involved.

DIAGNOSIS: The diagnosis of ano-rectal injuries, is easy, when the nature of the accident has been learned, the degree of hemorrhage, bruising and swelling have been noted, and the buttocks, anus, and rectum have been inspected, and digitally and proctoscopically examined.

TREATMENT: Minor injuries take care of themselves, while extensive injuries may require simple or complicated treatment.

Incised wounds are sutured, under aseptic conditions.

Contused, lacerated and pneumatic injuries are drained at one or more points, following irrigation, and the removal of ragged edges and necrotic tissue. Subsequently they are treated by drainage and topical applications, as fistula wounds.

Injuries of the bladder and urethra are immediately closed by when feasible, but if not, the bladder is drained, and the wounds here and in the rectum are permitted to heal by granulation.

Small recto-vesical rents are sutured, but where the rectum or Sigmoid is extensively injured, the bowel is resected, or an artificial anus is established.

Recto-vaginal tears, are repaired by suturing the vaginal before the rectal side of the wound is closed.

### THE CONSIDERATION OF RECTAL AND COLONIC DISEASE IN LIFE INSURANCE EXAMINATIONS.

By Alfred J. Zoebel, M. D.,

Fellow of the American College of Surgeons; Chief of Department of Rectal Surgery, San Francisco Polyclinic and Post Graduate School, San Francisco, California.

All important data concerning the vital organs is obtained by a medical life insurance examiner by direct examination and by precise methods. On the other hand, life insurance companies evidently do not attach much importance to the condition of the rectum and colon—not to mention the rest of the alimentary canal—for they seem willing to assume that these organs are free from disease solely from the favorable answers given by the applicant to routine printed questions asked by the examiner. That this is a fallacy, inasmuch as it paves the way to the acceptance of poor risks, and occasionally to the rejection of a good one, is shown in this paper.

Applicants almost invariably deny having or ever having rectal or colonic disease. The writer thinks that perhaps the main reason for this general denial is the ease with which these affections can be concealed from the examiner, unless he makes an examination.

The average individual knows little about his ano-rectal region, and unless there is severe pain or itching, alarming bleeding, or annoying dysentery, he thinks it of little importance and unworthy the attention of either himself or the examiner. The rectal surgeon often sees individuals who look and feel in the best of health (outside of "a little attack of pile"), yet who are found victims of well advanced malignant disease of the rectum or colon. Unless a rectal examination be made such a person could easily pass a life insurance examination.

The examiner should look out for those little fistulous tracts which cause no pain and discharge but little secretion, as they are frequently the primary manifestations of tuberculosis, and may appear in those who are otherwise apparently healthy. A severe stricture of the rectum may be present in a man outwardly perfectly healthy and insurable. If no history of his condition was volunteered such a person could pass an examination unless the rectum was examined.

If a history of hemorrhoids is secured, or if on examination, it should not be forgotten that although their existence does not constitute a good cause for rejection, they often accompany liver, spinal cord, genitourinary and uterine disease.

In cases where a suspicious anemia is found to be due solely to bleeding from hemorrhoids, these individuals could be conserved to the life insurance business if put in the way of regaining their health so as to become insurable.

If a rectal examination is made the condition of the genito-urinary organs in the male can be investigated at the same time, while in the female accurate information can be obtained about their plevic organs without subjecting them to a vaginal examination. At the present time insurance companies do not demand an examination of the female generative organs but accept their answers to the questions whether they ever had any uterine disorder, and if pregnancy now exists.

In conclusion, the suggestion is offered that medical examiners should lay more stress upon the questions regarding the condition of the bowel and rectum. They should enquire carefully whether there is or has ever been a sanguinous, purulent or mucous discharge from the rectum. A history of chronic constipation or of diarrhoea should be consideed worthy of further investigation. A rectal examination, both digital and instrumental, should follow if there is need therefor, or whenever there is the slightest suspicion that by it something may be revealed.

That medical examiner is the most "efficient" who not only secures his company from poor risks but also saves its business which otherwise would be lost. The utilization of rectal examination helps attain "efficiency."

### SPASMODIC STRICTURE OF THE RECTUM.

By Louis J. Krouse, M. D., F.A.C.S., Cincinnati, O.

Dr. Krouse says that spasmodic stricture of the rectum was called phantom stricture on account of its imaginary existence.

He makes the statement that in the early part of the last century it was more frequently diagnosed than later on. At the present time, the opinion regarding the existence of such an affection is equally divided between those who are firm believers and those that doubt its existence.

After quoting the statements of various authors well versed in rectal pathology, he expresses his own opinion in its existence and reports several cases. He also makes the statement and agrees with a few writers who believe that spasmodic stricture is often the forerunner of the more serious disease of benign stricture of the rectum. He reports several cases.

He claims that spasmodic stricture is not a disease but only a symptom of some other disease located in the rectum or in an adjoining organ.

His conclusions are:

First: That it is not a common affection.

Second: That it is easily detected on digital examination.

Third: That it often terminates in an annular fibrous stricture.

Fourth: That it involves the lower Houston valve.

Fifth: That a rectal ulcer is the most important etiological factor.

Sixth: Curing the ulcer in its early stage lessens the chances of the development of an annular fibrous stricture.

Syphilis regarded as a contagious disease as other exanthemata is characterized by its chronicity and virulency. The only exception to its point of inoculation being confined to tissures covered by squamous epithelim, is within the rectum.

Its frequency in the rectum and anus is not realized and, in consequence is not recognized by the profession. Its relationship to fistulae and stricture is emphasized, and the importance of tuberculosis in these two conditions minimized. The successful treatment of fistulae is proverbial. The possibility of stricture, resulting from secondaries later in life, suggested.

### ABSTRACT—ACUTE ANGULATION AND FLEXURE OF THE SIGMOID A CAUSATIVE FACTOR IN EPILEPSY—REPORT OF NINE NEW CASES WITH FOUR RECOVERIES.

By W. H. Axtell, M. D., A. M., Bellingham, Washington.

Review: In December, 1910, I published my first list of thirty-one cases—eight private and twenty-three Asylum cases; in August, 1911, a further report on ten private cases with three recoveries—this included three additional Asylum and two private cases, making in all thirty-six cases. The three reported cured have remained so for a period now of over four years. One additional case (No. 4) of the original list of ten private cases has had no return of the convulsions since ceasing treating two years ago, treatment seemed at the time to increase the irritation as reported.

Additional Cases: Since last report I have had nine additional cases with four of them remaining free from seizures for from one year to two and a half years, making in all forty-five cases reported with eight recoveries to date.

Observation: From my observations I am convinced that those who acquire epilepsy after the fifteenth year are more amenable to successful treatment than when commencing earlier in life. In my judgment surgery can give but little relief except where there is a definite history of inflammatory adhesions holding the angulations and flexures,—in fact, the condition of fecal stasis precludes surgery of the colon until the condition is first relieved, which when so relieved a prime factor in the production of the trouble is eliminated. A new and undescribed cause of the intestinal ptosis which is so generally present in these cases is the separation of the Recti Muscles, which are so essential to a thorough evacuation of the colon and for the support of the abdominal organs.

The essential failure of treatment of these conditions lies in the fact that so few recognize the true condition, and, if the condition is recognized, there is not sufficient persistence in relieving the condition, or an ignorance as to the amount of material the colon holds and as to when it is well emptied, that is the reason so many fail and as a result mutilating surgery is resorted to without getting results commensurate to the gravity of the surgery resorted to,—the first intimation of the true condition is found upon opening the abdomen,—then details are carried out which should have been used in the first instance, then surgery would be unnecessary.

### THE RELATION OF THE ROENTGENOLOGIST TO THE PROCTOLOGIST.

By Walter I. LeFevre, M. D., Cleveland, Ohio.

This paper calls attention to the advancement made in Roentgenology in recent years, and gives statistics as to the men devoting their entire time to the subject. He also mentions the increase of special literature upon the subject, as well as the immense manufacturing interests which have sprung up.

The conclusion is drawn that to the proctologist the x-ray is of value just in proportion as he is interested above the sigmoid flexure. Below this point the proctoscope gives direct information.

Because of the expense and the refinements of technic the writer feels that the proctologist should work in conjunction with his friend the Roentgenologist.

### POSITION FOR SIGMOIDOSCOPIC WORK.

By Donly C. Hawley, A. B., M. D., Burlington, Vermont.

A majority of writers express a preference for the knee-chest position, while a minority prefer some other, e. g.: Hanes, Sime, or the exaggerated lithotomy position.

Before the days of the pneumatic sigmoidoscope the position was of necessity such as would admit of inflation by atmospheric pressure. Here the knee-chest position was undoubtedly the most satisfactory.

The knee-chest position is trying and disagreeable for the patient and not easy nor always convenient for the operator.

Its use is frequently attended with embarrassment and fear on the part of the patient.

With the pneumatic tube the older method may be done away with.

Place patient in left lateral prone position with left arm drawn out behind back, the patient lying well over on left chest and stomach, the knees flexed, the right more than the left and placed above and well over and beyond the left on the table and with the back concaved as much as possible.

In this position the abdominal muscles are relaxed, while in the knee-chest position they are apt to be contracted.

In a majority of cases the instrument may be passed easily and quickly over the brim of the pelvis and into the sigmoid colon as far as required or to its full length.

This method not advocated exclusively, but a more thorough trial is urged.

### TUBERCULOSIS CUTIS ANI.

By D. C. McKenney, M. D., F. A. C. S., Buffalo, N. Y.

An interesting case of tuberculosis of the anal skin is reported.

From the clinical study of the case Dr. McKenney infers that the injection started from the anal canal rather than in the skin around the anal orifice. An active respiratory infection, associated with aphonia, seems strong evidence that the injection was carried in the feces to the anus. Two photographs of the local condition were presented.

### A BRIEF REPORT OF TWO CASES OF ANAL HERPES ZOSTER.

By Lewis H. Adler, Jr., Philadelphia, Pa.

Dr. Lewis H. Adler, Jr., stated that cutaneous legions about the anal region are by no means unusual, and that the frequency of their occurrence is much less than one might reasonably expect from the function of the part,—its more or less constant contact with germ-laden feces; the frequent congestion to which it is subjected and the attrition of the nates and adjacent structures induced by walking, etc.

That in this connection a very unusual condition, so far as his experience went, was Anal Herpes Zoster, of which he had only seen two cases in his practice, both being in young women, —one of whom thought she had contracted some venerable trouble from using towels in a public bathing establishment.

That in both instances, the eruption was preceded for several days by a mild febrile disturbance, with burning pains in the anal region; at times the sensations were neuralgic in character. That in both patients, the lesions were confined to a definite area, affecting only one side of the anal cutaneous surface; that the eruption in neither case was very extensive nor numerous and there was no history of previous attacks or of similar touble elsewhere.

That the vesicles in both cases followed the usual course if Herpes Zoster occuring elsewhere,—the liquid they contained was clear, translucent serum, at first; which gradually became cloudy and later puiform. That they never evinced any tendency to rupture and in the course of ten days or two weeks, they gradually dried to thin yellowish or brownish crusts, which shortly dropped off,—after which there was left a reddish spot, covered wit hthe epidermis; and that these spots were vey slow in disappearing.

That the local discomfort in both cases was not lessened on the appearance of the eruption: but more or less burning was experienced, until the eruption had practically disappeared and that in one case it continued for several weeks afterward.

That the pain was so severe, in one case, that family physician found it necessary, on several occasions, to prescribe an anodyne.

That the treatment in each case was similar,—internally, liquor potassi arsenate, six drops was prescribed, locally, the parts were cleansed with a two per cent. creoline solution and freely dusted over with borated talcum powder. Over this a wad of absorbent cotton was applied and kept in place by an appropriate bandage.

### ABSTRACT.

Dr. William H. Kiger, Los Angeles, California, reports six cases of Pruritis Ani treated by the vaccine method as suggested by Murray. Cultures were taken from the skin at the anal junction. In every instance, Streptococcus Haemolyticus found. No local application of any kind was used. The results are attributed to vaccine treatment alone. He discredits the use of stock vaccines, and suggests the use of Autogenous Vaccine only. Considers the focal infection as a prime factor in an etiologic way. Also he reports three cases evidently due to an infection from abscesses at the roots of teeth. He says that all of the cases reported had Pyorrhea, and suggests a thorough examination of the teeth, together with an x-ray picture of the jaw. He believes that a re-infection often takes place.

---

### GAS POISONING—OBSERVATIONS DURING A MEDICAL SERVICE WITH THE BRITISH EXPEDITIONARY FORCE IN FRANCE.

Ernest T. F. Richards, M. D., St. Paul, Minn.
(St. Paul Medical Journal.)

An opportunity to study the clinical and pathological effects of "poisonous gas" was afforded the writer while serving last winter (1915-1916) with the British Army Medical Service in France. During the service a great many "gassed" cases were observed; in the course of a single night, for example, over one hundred victims of this appalling method of warfare were admitted to the medical wards.

The gas employed by the Germans is now commonly held to be chlorine. Its greenish-yellow color, its pnngent odor, its extremely irritating effect upon all mucous membranes, the relative ease of its manufacture, and its weight (two and one-half times heavier than air) go far to support this view. Leonard Hill (1) has pointed out that poison gases were investigated in Germany for years by Lehmann and his pupils from the ostensible point of view of making safe dangerous trades. From a critical survey of Lehmann's papers, Hill believes the conclusion is inevitable that if any gases were used in warfare they would be chlorine or brimone. They alone come up to the requirements, namely, (1) that a 1 to 10,000 concentration rapidly puts a man out of action—by asphyxiating him, owing to its intense irritative property; (2) they are much heavier than air; (3) they are manufactured in huge quantities in trade processes; (4) they are easily compressible into cylinders for convenience of transport and handling. Moreover, a respirator is easily contrivable to protect the person who manipulates the brigade gas attack; and it is obvious that no drift gas can be used offensively from which the users are unprotected.

PATHOLOGY.

Both experimentally and from autopsies on subjects dead of gas poisoning much has been learned concerning the morbid anatomy of the condition. Using rabbits, Sir Edward Schafer (2) found that with a mixture of one part of chlorine gas to twenty of air, and with all in excess of this, a fatal result was rapidly and inevitably produced. He believes the effects which might be produced locally in the lung by an irritant gas like chlorine are various. It may directly affect the bronchial musculature or the vascular musculature; it may stimulate the mucus-secreting mechanism of the air tubes; it may influence the coagulability or viscosity of the blood within the pulmonary vessels; or it may produce reflex effects by exciting the endings of different nerves within the lungs. Schafer concludes there is strong evidence for the opinion that the fatal result is due to obstruction in the pulmonary vessels rendering it impossible for the blood to pass freely to the left auricle and ventricle.

Hill (1) has well summed up the pathological findings in individuals dead of gas poisoning. "Post-mortem examination in the acute cases shows an intense congestion of the mucosa of the trachea and larger bronchi. These tubes are filled with a thin, light yellow frothy secretion, some of which escapes from the mouth and nose when the cases are first laid on the table. The fluid is highly albuminous, solidifying on heating. The larger bronchi only can be traced, the smaller being lost in a condition of intense congestion and edema which affects the lungs as a whole. The lungs do not collapse in these acute cases, but appear like a solid cast of the thoracic cavity, and are greatly increased in weight. On incision, the lung tissue appears of a deep maroon red color, and the exudation flows from the cut surfaces in abundance. Light grey patches are to be seen on the surface of the lungs amidst the congested areas. They were found to be due to emphysema. So intense is the obstruction to the entry of air, and so violent the efforts of respiration, that emphysema is produced in these least poisoned parts where air can still enter. We can picture how the violence of the respiratory efforts, brought to bear on a relatively few small parts of the lungs, distends and breaks down the walls of the alveoli, expelling the blood into surrounding congested areas. The parts of the lung tissue not affected by emphysema show the intense congestion of the capillaries, and many of the alveoli are seen filled with exudate which takes on the eosin stain. Into some alveoli red corpuscles escape; larger patches of haemorrhage may occur. The heart in these acute cases is congested, particularly on the right side. The stomach shows a condition of catarrh, the mucosa being covered with a thick yellowish mucus and haemorrhages being visible in the submucosa. These changes may conveivably be due to the swallowing of saliva and exudate from the nose and expectorated fluid in which chlorine is dissolved. The venous congestion of the stomach and other abdominal organs and of the brain is due to the asphyxial character of the death."

### SYMPTOMS.

The extent to which an individual is affected by the poisonous fumes differs somewhat, depending largely upon how securely he is able to protect himself with his gas helmet at the time of the attack. The use of these helmets has undoubtedly cut down the mortality rate from gas attacks materially. Both the British and French soldiers place great faith in them as a means of protection, and many of them stated that if they had had their helmets on in time, or if the masks had not been torn, they believed they would have escaped poisoning. In those most seriously affected the picture of suffering presented almost beggars description, but the main clinical symptoms may be classified as follows:

Cyanosis; dyspnea; cough with profuse expectoration; pain in the chest; irritation of the throat; salivation; conjunctivitis; stupor; headache; eqigastric pain, nausea and vomiting; fever.

Cyanosis was one of the most striking features. Affecting chiefly the face, hands and feet, it approached in the severely poisoned a deep, diffuse, blue-black color. Curiously persistent, it was one of the last evidences to go in a convalescence always very prolonged. A further characteristic was its tendency to recur on slight exertion; many of the patients upon being allowed to sit up after being in bed for weeks would become so cyanotic that another period of rest was imperative.

Dyspnea of varying intensity, depending upon the severity of the case, was always present. Frequently it was all that extreme "gasping" type audible all over the ward. Partial relief in such instances was obtained only by propping up the patient in a sitting posture with pillows, or having him lean forward on the side of the bed.

Cough was a prominent symptom, and ranged from the short, irritative type to the severe paroxysmal, in which the distress of the patient reached an extreme degree. In the course of three or four days, the cough usually became productive and, as the condition progressed, was accompanied by a profuse mucopurulent expectoration. The amount expectorated per day was frequently very large. An interesting feature of the expectoration was, that upon microscopic examination, it was almost entirely free from micro-organisms and leucocytes, being made up almost wholly of epithelial cells.

Pain in the chest, chiefly referred to the substernum, and associated with a sense of constriction, was one of the chief complaints. During coughing paroxysms it was greatly aggravated and frequently necessitated an opiate.

Irritation of the throat, sometimes associated with excoriations of the buccal and pharyngeal membranes; and salivation of varying degree, existed in practically all cases.

Conjunctivitis was usually slight, but was often accompanied by photophobia which added materially to the distress of the patient.

Stupor in the severely poisoned approached a state of semi-coma, in which the patient lay obvious to his surroundings and only with great difficulty could be aroused to take nourishment. Accompanying the stuporous condition, diminished sensibility of the skin to pain and touch, and dulling of the deep reflexes were usually found.

Headache was usually a prominent complaint. Generally of a dull, diffuse character, it was often intractable, persisting frequently with great obstinacy.

Epigastric pain in many occurred immediately after exposure to the gas and was apparently due to the swallowing of some of the irritant. It was accompanied by nausea and frequently by vomiting, which latter, in many instances, gave relief. In some, distres in the upper abdomen, with a tendency to be easily nauseated, persisted for many days and made the nourishing problem a difficult one.

Fever was practically a constant finding and existed to some degree even in those milder types, which, in other ways, showed only slight effects of the poisoning. In certain of the severely toxic cases it attained a height of 103 degrees during the first two or three days. Usually it was of a rather low (100 degrees) remittant character, but was curiously persistent, remaining in several patients even after four or five weeks' stay in the hospital.

### PHYSICAL SIGNS.

"Gassed" patients upon being admitted to the hospital presented, in most instances, a degree of collapse, which, with the stuporous condition shown by most of them, made coherent accounts of their experiences almost impossible. The cyanosis, with the evidences of irritation of the visible mucous membranes has already been commented upon.

Upon examination, the findings in the chest were by far the most prominent.

Lungs: Difficult, rapid respiration and pain upon breathing produced in the typical case a very evident diminished chest expansion. Over both lungs, but most marked over the lower lobes, there was present an impaired note of resonance ranging from slight to perceptible dullness. Throughout the chest, frequently obscuring the heart tones, harshened breath sounds, accompanied by rales of every known variety, were the prominent findings upon auscultation. Among the rales, especially notable were the coarse, bubbling type, situated at the lung base particularly; and the generally diffused sibilant rale. In about two per cent. of our cases definite broncho-pneumonic patches of consolidation appeared at the end of the first week of illness. In one case of the series, a pleuritis serous exudate, complicated the pulmonary picture in the fourth week.

Heart: With the respiratory mechanism being taxed to its utmost, giving of the myocardium might well have been expected, but a striking absence of definite physical signs of cardiac dilatation—except in one or two isolated instances—was one of the most interesting features of this study. An accelerated heart rate, frequently ranging from 110 to 140, was, however, an invariable finding. The tones of the heart, too, were usually enfeebled. These features, together with lowered systolic blood-pressure readings (ranging from 95 to 110) and the symptomatic evidences already noted, made the probability of a myocardial insufficiency in the majority of cases fairly certain. In a few instances, upon subsidence of the marked pulmonary signs, a short systolic blow became audible over the mitral area.

Other physical findings were: a heavily coated tongue, often of a greenish-yellow character; foul breath; tenderness in the upper abdomen, being in some patients very marked; diminished pupillary, skin and deep reflexes, depending upon the depth of the stupor.

Broadbent (3) has drawn attention to nephritis as one of the deadly sequelae of gas poisoning.

COURSE OF ILLNESS.

In the majority of our cases, even in those classified as mild, both physical signs and symptoms were very slow in clearing up. This was especially true of the cyanosis, which showed great tardiness in disappearing and a marked tendency to return upon slight exertion, such as sitting up in bed, even when all other evidences of poisoning had gone. Dizziness was another obstinate symptom and often while not present with the patient lying in bed, was frequently very troublesome during convalescence when attempts were being made to get the patient up and around. In several cases, owing to the dizziness, confinement to bed was necessary during the entire stay in the hospital—which extended sometimes to five and six weeks, after which, if not sufficiently well to be transferred to a convalescent camp, the patients were sent to England by hospital ship. Cough and expectoration were also slow in improving and persisted in many cases well into convalescence. Fever of slight or moderate degree lasted in the most severely ill throughout their entire stay in hospital. Clearing up of the lung findings, we noted, practically always tool place first in the upper lobes, the lung bases being the last to become free of rales.

Convalescence, even in mild cases, was slow and attended with considerable muscular and nervous exhaustion. Several patients, after a five weeks' stay in bed, had to be shipped on stretchers to England to convalesce. Recurrence of cyanosis and dizziness on exertion, coupled with a low blood pressure and an accelerated pulse, frequently made convalescence a circulatory problem.

TREATMENT.

No standard treatment of this distressing condition can yet be said to be established. Watt and Irvine (4) found emetics (copper sulphate and apomorphine) to be of value in the early stages; and later, as the lungs became edematous, blood letting was of apparently distinct benefit. Symes, (5) from experimental observations on cats, believes that the respiratory obstruction set up by inhalation of irritant gas is relieved by inhalation of stramonium and other fumes. Mortimer (6) draws attention to subcutaneous injections of oxygen being followed by relief of the respiratory obstruction. Black, Glenny and McNee (7) employed as their routine treatment: abundant supply of air and warmth; an emetic of salt and water if the patient was very cyanosed and had not already vomited, followed by ammonium carbonate and vinum ipecacuanhae; oxygen inhalation in cases of marked cyanosis and dyspnea; opium in restless cases to allay mental strain; and pituitary extract (1 ccm.) and brandy when the heart threatened to fail.

The treatment adopted in our series of cases consisted of: Rest in bed; fresh air; forced nourishment; oxygen inhalations; venesection; atropine sulphate; strychine, digitalis, and morphine.

It is somewhat problemental which of these measures were of the greatest moment, but with a large number of cases desperately ill on admission the total mortality rate under this regime was only 3.5 per cent.

Rest in bed with all windows wide open, the patient being kept as warm as possible, and forcing of nourishment were undoubtedly important factors in saving the lives of many.

Inhalations of oxygen strikingly relieved the air hunger, the cyanosis and the distressing dyspnea. In those most seriously ill it was freely used at short intervals, the patients themselves frequently begging for it.

In a few cases where, owing to extreme cyanosis, greatly labored breathing, or the physical findings of right heart dilatation, it was deemed necessary to mechanically relieve the embarrassed circulation, venesection was employed. This was followed in each instance by a distinct lessening of the cyanosis, diminution of the headache, and improvement in the other symptoms. From our observations, it would appear that this measure to be of most value should be carried out at rather frequent intervals, with the withdrawal of only moderate or small amounts of blood on each occasion.

Of the drugs used, atropine sulphate, hypodermatically administered, was of definite value, particularly during the stages of great pulmonary edema. It is to be especially noted that, in order to be effective, this drug must be employed in maximum doses at frequent intervals. Strychnine was of doubtful, but in a few instances of some apparent value. Digitalis, after the acute stages of the poisoning had subsided, and particularly during convalescence, was found to exert a distinctly beneficial effect.

REFERENCES.

1. Hill, Leonard: Brit. Med. Jour., No. 2866, 1915.
2. Schafer, Sir Edw.: Brit. Med Jour., No. 2850, 1915.
3. Broadbent, Walter: Brit. Med. Jour., No. 2850, 1915.
4. Watt, Andrew H. and Irvine, Louis G.: Brit. Med. Jour., No. 2850, 1915.
5. Symes, W. L.: Brit. Med. Jour., No. 2844, 1915.
6. Mortimer, J. D.: The Lancet, London, Vol. clxxxviii, No 4789, 1915.
7. Black, Glenny, and McNee: Brit. Med. Jour., No. 2848, 1915.

